# Emission Enhancement of Cu-Doped InP Quantum Dots through Double Shelling Scheme

**DOI:** 10.3390/ma12142267

**Published:** 2019-07-15

**Authors:** Hwi-Jae Kim, Jung-Ho Jo, Suk-Young Yoon, Dae-Yeon Jo, Hyun-Sik Kim, Byoungnam Park, Heesun Yang

**Affiliations:** Department of Materials Science and Engineering, Hongik University, Seoul 04066, Korea

**Keywords:** Cu doping, InP quantum dots, double shelling, emission enhancement, electroluminescence

## Abstract

The doping of transition metal ions, such as Cu^+^ and Mn^2+^ into a quantum dot (QD) host is one of the useful strategies in tuning its photoluminescence (PL). This study reports on a two-step synthesis of Cu-doped InP QDs double-shelled with ZnSe inner shell/ZnS outer shell. As a consequence of the double shelling-associated effective surface passivation along with optimal doping concentrations, Cu-doped InP/ZnSe/ZnS (InP:Cu/ZnSe/ZnS) QDs yield single Cu dopant-related emissions with high PL quantum yields of 57–58%. This study further attempted to tune PL of Cu-doped QDs through the variation of InP core size, which was implemented by adopting different types of Zn halide used in core synthesis. As the first application of doped InP QDs as electroluminescent (EL) emitters, two representative InP:Cu/ZnSe/ZnS QDs with different Cu concentrations were then employed as active emitting layers of all-solution-processed, multilayered QD-light-emitting diodes (QLEDs) with the state-of-the-art hybrid combination of organic hole transport layer plus inorganic electron transport layers. The EL performances, such as luminance and efficiencies of the resulting QLEDs with different Cu doping concentrations, were compared and discussed.

## 1. Introduction

Colloidal semiconductor quantum dots (QDs) have been regarded as promising emitters for the next-generation display and lighting devices in the light-emitting diode (LED) platforms of color-conversion and electroluminescence (EL). Photoluminescence (PL) of QDs with a given composition is systematically tunable by virtue of synthetically controllable size variation. In addition, impurity doping into QDs is another means to modulate their PL, while their band gap remains by and large unaltered. In this regard, doped QD emitters possess an intrinsically large Stokes shift, by which self-absorption can be effectively suppressed. Mn^2+^ is one of the most common impurity ions for doping QDs with different host compositions such as CdS [1,2,3], ZnSe [4,5,6,7], and ZnS [8,9]. One principal feature of Mn-doped QDs is the insensitiveness of their PL energy to the composition and size of the QD host, since the radiative recombination of Mn^2+^ ion excited through the transfer of exciton energy of host QD is governed by the *d* orbital intrashell electronic transition of ^4^T_1_–^6^A_l_. Cu^+^ is another popular transition metal ion for doping into various QD hosts including the above II–VI chalcogenides adopted for Mn doping [10,11,12,13,14]. Distinct from Mn^2+^ emissive channel, the radiative transition of Cu^+^ dopant results from the recombination of an electron in a conduction band with a hole localized at Cu^+^ *d*-orbital state. Taking into account that the energetic position of Cu^+^ state is pinned in a given host composition relative to a vacuum [15] and the energy level of conduction band is movable as a function of QD size, tunable emissivity is expected from Cu-doped QDs, specifically yielding a longer wavelength in PL from a larger QD size.

Further, III–V InP QDs are emerging visible emitters particularly for the application to display devices owing to their low-toxicity (i.e., Cd-free composition) as well as high-quality fluorescent attributes (e.g., high PL quantum yield (QY) and narrow PL bandwidth). II–VI metal chalcogenides with an ionic bonding character may be a better host for Cu doping than III–V InP with a stronger covalent bonding (i.e., weaker bond breaking ability) [16]. Nevertheless, successful Cu doping in InP host QDs, albeit limited, has been demonstrated typically by either nucleation doping [17] or the surface adsorption-lattice diffusion approach [16,18,19]. Among a few reports on Cu-doped InP (InP:Cu) QDs, Mei et al. claimed that dual emission in a single InP:Cu QD was obtainable from the combined PL of two competitive excitonic and Cu state-related recombinations [17]. When the impurity as a luminescent center is properly doped in a certain QD host, however, the complete quenching of excitonic PL is expected with dopant-associated PL exclusively emerging, since the trapping rate of photoexcited charge carrier at dopant state is much faster than the excitonic recombination rate [7,15]. In this context, dual emission observed in doped QDs alludes to the unwilled coexistence of undoped plus doped QDs in the ensemble. All InP:Cu QDs reported to date possessed a simple core/shell heterostructure with a single shell of either ZnSe [18] or ZnS [16,17]. From the state-of-the-art undoped InP QD systems, however, a single shelling in particular with ZnS is not an ideal scheme in securing high fluorescence, since a large lattice mismatch (ca. 8%) between InP and ZnS induces a considerable interfacial strain at core/shell, deteriorating PL efficiency. Therefore, to relieve the interfacial strain, an intermediate (or inner) shell typically with the compositions of GaP [20] and ZnSe [21,22,23] has been inserted prior to ZnS outer shell. In the same context, the introduction of such double shelling scheme to doped QD systems is expected to improve dopant-related PL.

Parallel with synthetic advances of QDs, QD-EL (also called QLED) devices have been intensively explored for last two decades. The device performances of QLEDs with respect to luminance and efficiency are now in close proximity to those of organic LEDs. In such high-performance QLEDs, however, high-toxicity CdSe QDs have been leading materials. On that account, a great deal of effort has been dedicated to the development of high-performance QLEDs based on non-Cd emitters, such as InP [23,24,25], ZnSe [26,27] and Cu–In–S QDs [28,29]. To date, the fabrication of QLEDs has been carried out exclusively with undoped QDs regardless of QD composition, while EL work on doped QDs remains nearly unexplored. In this work, the authors report on a two-step synthesis of highly fluorescent InP:Cu QDs, where Cu doping is carried out in the manner of surface adsorption-lattice diffusion and a double shelling scheme of ZnSe inner and ZnS outer shells is applied. The resulting InP:Cu/ZnSe/ZnS QD, the first doped QD system consisting of double shells with different compositions, exhibited the best PL QY of 57–58% at nominal Cu mole fractions of 0.05–0.1. This PL QY is the highest value reported to date among single dopant PL-capable InP:Cu QDs. The doping concentration and InP core size are individually varied to examine their effects on PL properties. The two representative InP:Cu QDs with different doping concentrations are for the first time employed as doped emitters for all-solution processed fabrication of multilayered QLEDs and their EL characteristics are addressed.

## 2. Materials and Methods

### 2.1. Synthesis of InP:Cu and InP:Cu/ZnSe/ZnS QDs

For a typical synthesis of InP core QDs, 0.3 mmol of indium iodide (InI_3_), 0.73 mmol of zinc chloride (ZnCl_2_) and 2 mL of oleylamine (OLA) were mixed in a 50 mL flask reactor, followed by degassing at 120 °C for 30 min and heating to 180 °C under N_2_ flowing. Then, 0.12 mL of tris(dimethylamino)phosphine (P(DMA)_3_) and 0.22 mL of trioctylphosphine (TOP) were swiftly injected into the above hot mixture and the growth of InP core proceeded at that temperature for 2 min. Further, a certain amount of Zn precursor was introduced in the present synthesis of InP core QDs. This was undertaken since its presence can not only improve size uniformity, but can facilitate the following shell growth [30,31]. Then, hexane and ethanol were added to precipitate the QDs, followed by centrifugation. These InP core QDs were dispersed in toluene for further use.

**Scheme 1:**
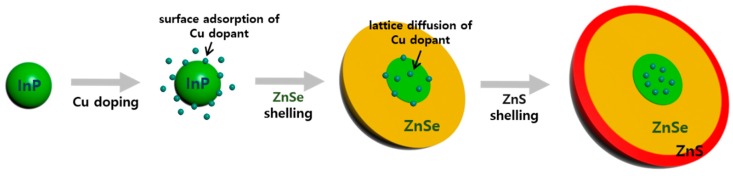
Schematic of synthesis of InP:Cu/ZnSe/ZnS quantum dots (QDs) by a surface adsorption-lattice diffusion strategy.

For Cu doping and the subsequent growth of double shells of ZnSe/ZnS, 2.2 mmol of ZnCl_2_, 3 mL of OLA, 3 mL of 1-octadecene (ODE) and the purified InP cores in toluene were loaded in a 50 mL flask. After degassing at 120 °C for 30 min to evaporate toluene, the mixture was heated to 130 °C. Furthermore, injected into this mixture was the copper precursor solution, consisting of 0.0067, 0.0167, 0.033 or 0.05 mmol of copper (II) chloride (CuCl_2_) in 3 mL of OLA, corresponding to nominal Cu mole fraction (i.e., Cu/(In + Cu)) of 0.02, 0.05, 0.1, or 0.14, respectively, followed by the reaction at that temperature for 1 min. Then, the reaction temperature was raised to 160 °C for the following shell growth. Anionic and cationic shell precursors were simultaneously injected for a stepwise growth of ZnSe inner shell as follows; Se-TOP solution (0.75 mmol of selenium (Se) dissolved in 0.75 mL of TOP) and Zn-ODE solution (0.79 mmol of zinc stearate dissolved in 2 mL of ODE) were introduced, followed by the reaction at 270 °C for 60 min. Subsequently, the Se-TOP solution (0.45 mmol of Se dissolved in 0.45 mL of TOP) and the same Zn-ODE solution as above were injected, followed by the reaction at 280 °C for 60 min. As the last step of ZnSe inner shelling, Se-TOP solution (0.3 mmol of Se dissolved in 0.3 mL of TOP) and the same Zn-ODE solution as above were injected, followed by the reaction at 290 °C for 60 min. For the consecutive growth of ZnS outer shell, the S-TOP solution (4 mmol of sulfur (S) dissolved in 2 mL of TOP) and the same Zn stock solution as above were added, followed by the reaction at 300 °C for 120 min. After lowering the temperature to 220 °C, 0.75 mL of 1-octanethiol (OTT) was introduced by a dropwise manner, and then the shelling was finalized by adding 1 mmol of Zn acetate dihydrate dissolved in 1 mL of oleic acid (OA) and the reaction was maintained at 190 °C for 120 min. The overall synthetic flow of InP:Cu/ZnSe/ZnS QDs is also provided in Scheme 1. After cooling to room temperature, as-synthesized QDs were precipitated by introducing excess ethanol, repeatedly washed with a solvent/nonsolvent combination of hexane/ethanol, and finally redispersed in hexane.

### 2.2. Preparation of ZnMgO Nanoparticles (NPs)

In a synthesis of ZnMgO NPs, 8.15 mmol of Zn acetate dihydrate and 18.5 mmol of Mg acetate tetrahydrate were dissolved in 40 mL of dimethyl sulfoxide (DMSO). Then, 10 mmol of tetramethylammonium hydroxide (TMAH) in 10 mL of ethanol was added dropwise in the above cationic precursor solution and the reaction was maintained at 4 °C for 1 h. As-synthesized ZnMgO NPs were purified with excess acetone and then re-dispersed in ethanol for the following use.

### 2.3. Fabrication of QLEDs

The 150 nm-thick indium tin oxide (ITO)-coated glass substrate was ultrasonic-cleaned with acetone and methanol for 20 min respectively, and then subjected to UV-ozone treatment for 20 min. Poly(ethylenedioxythiophene):polystyrenesulfonate (PEDOT:PSS, Al 4083) as a hole injection layer (HIL) was generated by spin-deposition at 3000 rpm for 30 s and baked at 150 °C for 20 min. Using 0.04 g of poly((9,9-dioctylfluorenyl-2,7-diyl)-*co*-(4,4′-(*N*-(4-s-butylphenyl))diphenyl-amine) (TFB) dissolved in 5 mL of chlorobenzene, 25 nm-thick hole transport layer (HTL) was produced by spin-casting at 4000 rpm for 30 s and baked at the same condition as in the above HIL. For the following emitting layer (EML) formation, two QD hexane dispersions of Cu-doped InP/ZnSe/ZnS with 0.05 and 0.1 Cu mole fraction, whose optical densities were adjusted to be 0.55 at 530 and 565 nm, respectively, were individually spin-casted at 2000 rpm for 20 s and baked 70 °C for 10 min. Then, on top of QD EML 40 nm-thick electron transport layer (ETL) was spin-deposited at 3000 rpm for 30 s by using ZnMgO NPs dispersion with a concentration of 38 mg/mL in ethanol and dried at room temperature. All spin-coating and baking processes in the formation from HIL throughout ETL were performed in a N_2_-filled glovebox. Finally, 100 nm-thick Al as a metal cathode was deposited by a vacuum thermal evaporation.

### 2.4. Characterization

The absorption and PL spectra of QDs were collected by UV-visible spectroscopy (Shimadzu, UV-2450, Kyoto, Japan) and a 500 W Xe lamp-equipped spectrophotometer (PSI Co. Ltd., Darsa Pro-5200, Suwon, Korea), respectively. The PL QY of QDs was evaluated in an integrating hemisphere with an absolute PL QY measurement system (QE-2000, Otsuka, Tokyo, Japan). The excitation wavelength for all PL and PL QY measurements was 450 nm. The transmission electron microscopic (TEM) work was performed using a JEM-2100F (JEOL Ltd., Tokyo, Japan) and a Tecnai G2 F20 (FEI Co. Ltd., Hillsboro, OR, USA) to obtain particle images of QDs and cross-sectional ones from QLED, respectively. The powder x-ray diffraction (XRD) patterns of QDs were collected using an Ultima IV (Rigaku) with Cu Kα radiation. The actual Cu concentrations of doped QDs were analyzed with an inductively coupled plasma optical emission spectrometry (ICP-OES, OPTIMA 8300, PerkinElmer, Waltham, MA, USA). PL decay profiles of undoped and Cu-doped QDs in the form of hexane dispersion were recorded using the time-correlated single-photon counting method employing a spectrophotometer (FS5, Edinburgh Instruments, Livingston, UK) equipped with a picosecond pulsed laser diode (EPL-375). The acquisition of EL data such as EL spectra and luminance-current density-voltage characteristics of QLEDs was carried out with a Konica-Minolta CS-2000 spectroradiometer (Konica-Minolta, Tokyo, Japan) coupled with a Keithley 2400 voltage-current source unit.

## 3. Results and Discussion

**Figure 1:**
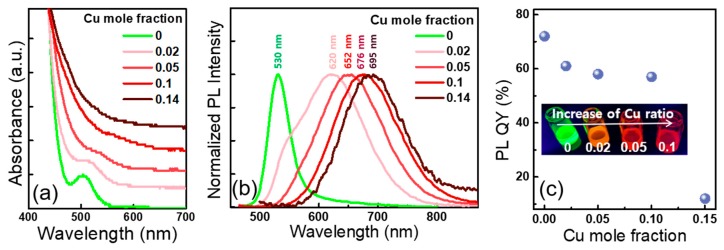
(**a**) Absorption; (**b**) normalized photoluminescence (PL) spectra; and (**c**) variation of PL QY of InP:Cu/ZnSe/ZnS QDs as a function of doping concentration of 0–0.14 Cu mole fraction. A fluorescent image of a series of QD samples under UV irradiation is shown in the inset of (**c**).

The present Cu doping was performed by surface adsorption and lattice diffusion, where Cu dopants were adsorbed on the surface of pre-grown InP core and incorporated into the core lattice through diffusion in the course of the subsequent shelling at an elevated temperature. Although the Cu (II) precursor was used for doping, the oxidation state of Cu dopant was expected to be +1 because of a high propensity to the reduction of Cu (II) to Cu (I) by electron-donating anions or ligands present in QD synthesis [12,17]. Figure 1a shows the variation of absorption spectra of double-shelled InP QDs with Cu dopant concentration. While undoped QDs exhibited a sharp absorption feature with an excitonic peak at 505 nm, the absorption peak of doped QDs became more indistinct and red-shifted with increasing Cu concentration. These spectroscopic changes likely indicate that Cu impurity plays double roles of electronic dopant [17,18] as well as luminescent center. Compared to a sharp excitonic PL (530 nm) of undoped QDs, Cu-doped ones yielded Stokes-shifted, broad PL bands as a result of Cu state-involved carrier recombination (Figure 1b). Upon doping with the lowest Cu concentration of 0.02 mole fraction, a minor spectral contribution from excitonic PL of InP QDs was observable, suggesting that a part of QDs in the ensemble remained undoped. When increasing Cu concentration to 0.05 mole fraction or higher, this unintended excitonic PL completely disappeared, showing a single band purely stemming from Cu dopant. In accordance with the absorption spectral evolution (Figure 1a), PL became systematically red-shifted with increasing Cu concentrations, being ascribed primarily to the electronic doping effect aforementioned. A high PL QY of 72% from undoped QDs dropped to some extent after Cu doping (Figure 1c). This signifies that Cu incorporation into InP host may adjunctively accompany the formation of lattice defects that serve as non-radiative recombination channels, being detrimental to PL QY. Nevertheless, high PL QYs of 57–58% were achieved from doped QDs with Cu concentrations of 0.05–0.1 mole fractions, which exhibited a pure Cu-dopant PL. These high PL QYs, with record values reported to date on doped InP QDs, are well attributable to the beneficial insertion of ZnSe inner shell. Its presence must effectively minimize the interfacial strain and consequent defect at the core/shell. This stems from a large disparity in a lattice constant between InP core and ZnS outer shell [21,22,23,25]. However, other doped InP QDs with Cu^+^ [16,17,18] or Ag^+^ ion [32] in the literature consisted of only a single shell. Although even higher PL QYs of 66 and 75% were reported from Cu- [17] and Ag-doped InP QDs [32], respectively, they commonly produced dual emissions of host excitonic plus dopant PL. Upon increasing Cu concentrations to 0.14 mole fraction, PL QY substantially decreased to 12% (Figure 1c). This may be understood by concentration quenching [32], higher probability of Cu^2+^ state population [17], or their combined effect.

**Figure 2:**
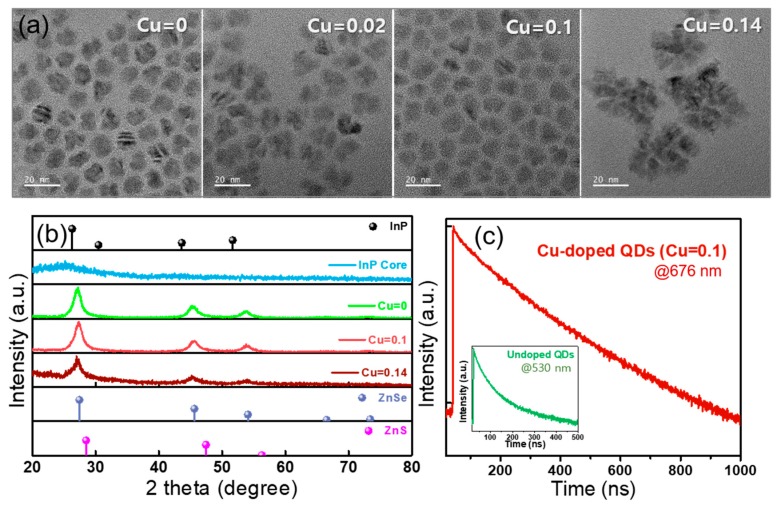
(**a**) TEM images of undoped and Cu-doped InP/ZnSe/ZnS QDs with 0.02, 0.1, and 0.14 Cu mole fraction. The scale bars are 20 nm for all; (**b**) XRD patterns of pure InP core, undoped and Cu-doped InP/ZnSe/ZnS QDs with 0.1 and 0.14 Cu mole fraction; (**c**) PL decay profiles of Cu-doped InP/ZnSe/ZnS QDs with 0.1 mole fraction and undoped QDs (inset) with detected wavelengths of 676 and 530 nm, respectively.

The TEM results in Figure 2a show that undoped, 0.05 and 0.1 (mole fraction) Cu-doped InP/ZnSe/ZnS QDs are by and large similar in size and particle morphology. The average sizes of the above three QD samples are distributed in 9.5–9.9 nm. These relatively large sizes (or thick shell thicknesses) were enabled by the inner shell of ZnSe having a small lattice mismatch (3.4%) with InP core. In the case of single shelled InP/ZnS QDs regardless of doping, their sizes were below 5 nm [16,17,33,34], since a large strain at InP/ZnS interface inhibited a thick growth of epitaxial ZnS shell. Meanwhile, Cu-doped QDs with 0.14 mole fraction were morphologically irregular, with smaller-sized particles agglomerated. This inhomogeneous growth may be caused by the excessive Cu ion adsorbed on the surface of InP core, which in turn hampered the uniform growth of the following ZnSe inner shell. In this context, the double-shell structure of such heavily doped QDs is expected to be slightly imperfect as those of its undoped and moderately doped counterparts. This incomplete heterostructure of 0.14 mole fraction Cu-doped QDs is also partly responsible for their poor PL QY. As compared in XRD patterns of undoped and 0.1 mole fraction Cu-doped InP/ZnSe/ZnS QDs (Figure 2b), the positions of three reflection peaks were identical regardless of Cu doping and its concentration. Their individual reflection peaks were shifted from those of InP to those of ZnSe and ZnS phases, indicative of a suitable formation of ZnSe/ZnS overlayers. Furthermore, nearly identical full-width-at-half-maxima (FWHMs) of the reflection peaks from undoped and 0.1 mole fraction Cu-doped QDs, indicative of their similar average sizes, are well in accordance with the above TEM results. Compared to undoped and 0.1 mole fraction Cu-doped QDs, broader FWHMs of the reflection peaks of 0.14 mole fraction Cu-doped ones were also consistent with their TEM results showing a smaller size. The actual Cu concentrations of a series of doped QDs, assessed by an ICP analysis, were below nominal ones, specifically showing actual Cu mole fractions of 0.004, 0.009, 0.016, and 0.02 relative to nominal values of 0.02, 0.05, 0.1, and 0.14, respectively. Thus, this indicated that the added fraction of Cu species participated in doping. Figure 2c presents a typical PL decay profile of representative Cu-doped InP/ZnSe/ZnS QDs with 0.1 mole fraction. Using a tri-exponential function fitting with the parameters of amplitude (A) and decay time constant (τ), i.e., *f*(t) = A1exp(–t/τ1) + A2exp(–t/τ2) + A3exp(–t/τ3), their average lifetime (τavg) was determined to be 281 ns (goodness-of-fit of 1.056), while the τavg value for excitonic PL of undoped QDs was 55 ns (inset of Figure 2c, goodness-of-fit of 1.105). A relatively long-decay behavior of Cu-doped QDs compared to undoped ones is characteristic of an intragap state-involved recombination, consistent with the values in the literature [16,17].

**Figure 3:**
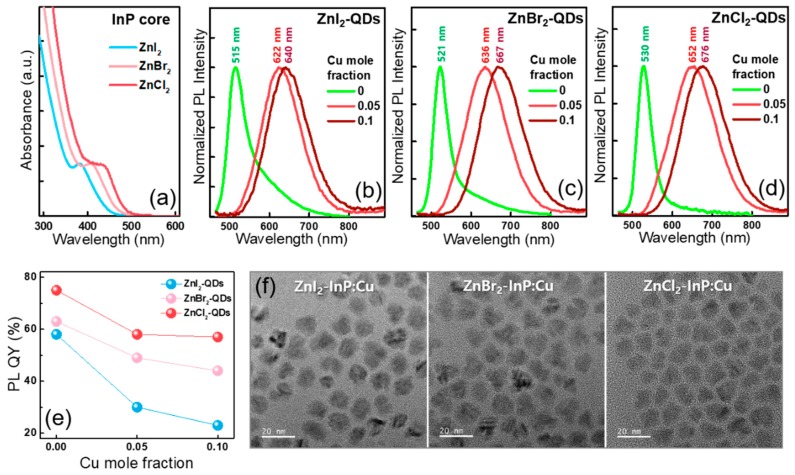
(**a**) Absorption spectra of InP cores synthesized with different Zn halide precursors of ZnI_2_, ZnBr_2_, and ZnCl_2_. The normalized PL spectra of undoped and Cu-doped QDs with 0.05 and 0.1 mole fraction based on InP cores synthesized with (**b**) ZnI_2_; (**c**) ZnBr_2_; and (**d**) ZnCl_2_; (**e**) The variations of PL QYs of a series of samples in (**b**–**d**); (**f**) TEM images of Cu-doped InP/ZnSe/ZnS QDs synthesized with ZnI_2_, ZnBr_2_, and ZnCl_2_ with the same Cu concentration of 0.05 mole fraction. The scale bars are 20 nm for all.

One of the most common strategies to tune PL of Cu-doped QDs is to vary the size of host QD [15,18,19]. For this, the host QD size is controlled by either growth time or temperature at a given reaction condition. Instead, this study attempted to control the size of InP QD by different types of Zn halide used in core synthesis with all reaction parameters unchanged. Zn chloride used in the original synthesis of InP QDs was replaced by other halides of Zn bromide (ZnBr_2_) and Zn iodide (ZnI_2_). In the case of aminophosphine-based InP QDs, the type of cationic precursors (i.e., In halides, Zn halides) affects their size, presumably since the steric hindrance of halide ions on adsorbed on QD surface differs by their size, eventually influencing the surface reaction rate [35]. Specifically, a tiny chloride ion relative to bromide and iodide may provide more available adsorption sites for growth, boosting the surface reaction rate towards larger QD sizes. Considering the size sequence of iodide > bromide > chloride ion, the smallest QD size is expected, if the synthetic condition is identical, from the use of Zn iodide. Figure 3a shows the comparison of absorption spectra of InP cores synthesized with ZnCl_2_ versus ZnBr_2_ and ZnI_2_. The excitonic absorption peak of InP core, an indicator of its band gap and consequently its size, systematically moved to the blue from 427 nm for ZnCl_2_-, 406 nm for ZnBr_2_- to 382 nm for ZnI_2_-based InP cores. This indeed verified that a more bulky halide ion generates a smaller-sized InP core. As compared in Figure 3b–d, the PL peak of undoped InP/ZnSe/ZnS QDs also gradually shifted from 530 for ZnCl_2_ to 515 nm for ZnI_2_, consistent with absorption spectral results (Figure 3a). Meanwhile, the PL of undoped QDs consists of two components of pure excitonic plus lower-energy tail emission. This persistent tail emission may be derived from a phosphorus dangling bond as a shallow trap state just above the valence band [36]. This tail emission became accentuated with decreasing size of InP core, alluding to more difficulty in effective surface passivation from smaller-sized QDs. Three InP cores with different sizes were then used for Cu doping with the representative concentrations of 0.05 and 0.1 in the mole fraction and PL properties of the resulting doped QDs were compared (Figure 3b–e). Analogous to the spectral trend in undoped QDs, the PL of doped ones showed a spectral shift to the blue from ZnCl_2_ (large-), ZnBr_2_ (medium-) to ZnI_2_ (small-sized InP core) at a given Cu concentrations, as expected. As summarized in Figure 3e, PL QY consistently decreased as the size of InP core became smaller, regardless of doping. This is generally explained by a higher difficulty in passivating a smaller-sized QD (aforementioned) with a higher surface-to-volume ratio.

As compared in the TEM images of three doubled-shelled QD doped with the same Cu concentration of 0.05 mole fraction (Figure 3f), all QD samples exhibited indistinguishably the same sizes of 9.1–9.3 nm. This was because a substantially thick shell thickness that primarily determined the overall QD size veiled the minute disparity in core size, even though the individual core sizes were indeed different.

**Figure 4:**
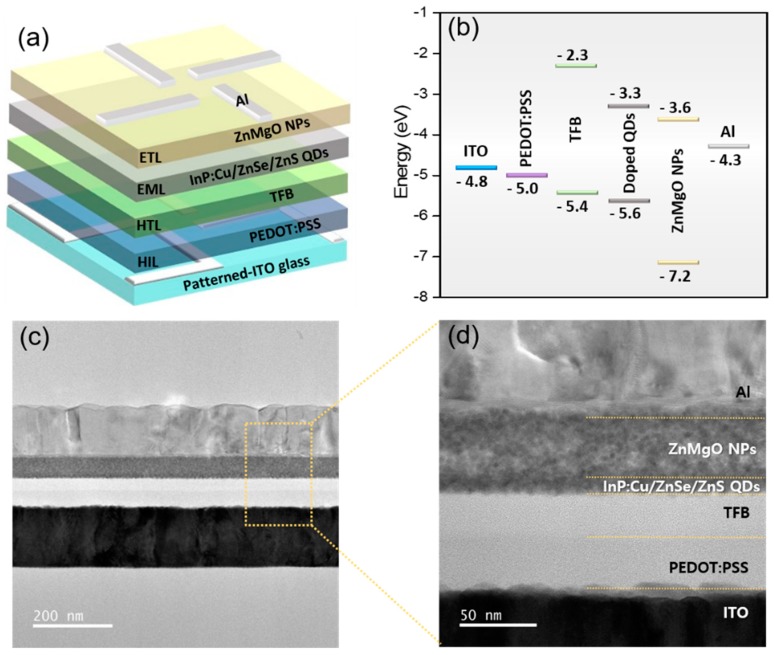
(**a**) Device schematic; (**b**) energy levels of individual layers; (**c**) low- and (**d**) higher-magnification cross-sectional TEM images of a multilayered quantum dot-light-emitting diodes (QLED). The scale bars in (**c**,**d**) are 200 and 50 nm, respectively.

**Figure 5:**
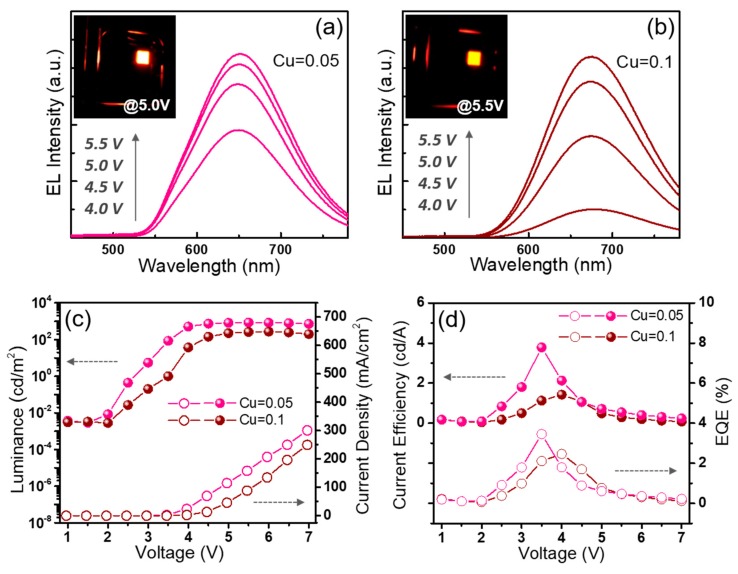
Voltage-dependent electroluminescence (EL) spectral evolutions along with representative EL images (insets) of QLEDs with Cu-doped QDs with (**a**) 0.05 and (**b**) 0.1 mole fraction. Voltage-dependent variations of (**c**) luminance and current density and (**d**) current efficiency and EQE of the same devices as in (**a**,**b**).

Almost all efforts on the fabrication of QLED devices has been made by using undoped QDs, while doped QDs have stayed nearly unexploited as EL emitters. Only a few doped QD-based EL devices were reported earlier based on CdS:Mn [2], ZnS:Mn [8], and CdS:Cu QDs [11]. In particular, red (615 nm)-emitting QLED with high-toxicity emitters of CdS:Cu QDs possessed the maximum external quantum efficiency (EQE) of 5.1% [11]. The ZnCl_2_-derived two doped QDs with 0.05 and 0.1 Cu mole fraction possessed high PL QYs of 57–58%. Herein, all-solution-processed QLEDs have been fabricated with a multilayered sequence of ITO // PEDOT:PSS // TFB // InP:Cu/ZnSe/ZnS QDs // ZnMgO NPs // Al (Figure 4a and also refer to Figure 4b for energy levels of individual layers of QLED). As seen from a cross-sectional TEM image of QLED with 0.05 mole fraction Cu-doped QDs (Figure 4c), individual functional layered were well defined without noticing inter-layer mixing. The layer thickness of QD EML was estimated by a higher-magnification TEM image (Figure 4d) to be ~15 nm, corresponding to approximately two QD monolayers. Figure 5a,b present typical deep-red EL spectra as a function of applied voltage of two QLEDs integrated with 0.05 and 0.1 mole fraction Cu-doped QDs, respectively. To the best of the authors’ knowledge, this is the first EL demonstration from Cu-doped InP QDs. EL spectral diffusion against the voltage increase was not observed, showing nearly invariant EL peaks at 650 and 675 nm for the former and latter device, respectively, which almost matched with their PL peaks (Figure 1b and Figure 3d). Both devices commonly yielded a single Cu dopant EL component without a parasitic spectral contribution from adjacent TFB HTL, pointing to the exclusive recombination of charge carriers injected at QD EML. As compared in Figure 5c, 0.05 Cu-based QLED exhibited higher current densities and a lower turn-on voltage than its 0.1 Cu-based counterpart. This was ascribable to the formation of a thinner QD EML of the former device compared to the latter one (which was experimentally verified from the comparison of EML thickness by scanning electron microscope measurements). Further, 0.05 and 0.1 Cu-based QLEDs produced maximum luminances of 861 and 269 cd/m^2^ at current density levels of 158 and 135 mA/cm^2^, respectively (Figure 5c). It is noted that a higher luminance from deep-red-emitting 0.05 Cu-based device compared to deeper-red-emitting 0.1 Cu-based one is attributable in part to the difference in the relative sensitivity of human vision-related photometric quantities including candela (cd) to a given emission spectrum. The maximum current efficiencies and EQEs were 3.8 cd/A, 3.5% and 1.4 cd/A, 2.4% obtained at the similar current density levels of 2.3–2.6 mA/cm^2^ for the former and latter device, respectively (Figure 5d). A higher EQE from 0.05 Cu-doped device relative to 0.1 Cu-doped one. Despite nearly the same PL QYs (57–58%) of both QDs are not clearly elucidated for the present, this may suggest that EL is more sensitive to Cu concentration quenching than PL, presumably associated with its relatively long decaying nature. By choosing 0.05 Cu-based device having better EL performances than 0.1 Cu-based one, the preliminary device lifetime test was implemented at ambient conditions after the conventional epoxy encapsulation. By driving a constant current density of 24 mA/cm^2^, corresponding to ~500 cd/m^2^, resulted in the half lifetime (T50) of ~0.5 h.

## 4. Conclusions

This study explored the synthesis of Cu-doped InP QDs with an elaborate double shelling scheme of ZnSe/ZnS. While the lowest Cu doping concentration of 0.02 mole fraction led to the minor appearance of excitonic PL from undoped InP QDs, increasing the Cu concentration to 0.05 mole fraction or higher gave rise to a single Cu dopant-related PL. PL QY reached the maximum values of 57–58% with optimal Cu concentrations of 0.05–0.1 mole fractions and then rapidly decreased to 12% upon a further increase to 0.14 Cu mole fraction. PL became gradually red-shifted in proportion to the Cu concentration, which the authors attributed presumably to the electronic doping effect of Cu^+^. For an additional effort to tune PL of InP:Cu/ZnSe/ZnS QDs, different-sized InP cores were synthesized by varying Zn halide precursors of ZnCl_2_, ZnBr_2_, and ZnI_2_ in core synthesis. As intended, a more bulky halide ion, i.e., iodide > bromide > chloride ion, produced a smaller-sized InP core, and thus PL of doped QDs exhibited a consistent spectral shift to the blue from ZnCl_2_ (large-), ZnBr_2_ (medium-) to ZnI_2_ (small-sized InP core) at a given Cu concentrations. By exploiting ZnCl_2_-based two doped QDs with 0.05 and 0.1 Cu mole fraction that showed comparably high PL QYs as EL emitters, all-solution-processed QLEDs were fabricated with a multilayered architecture of ITO // PEDOT:PSS // TFB // QDs // ZnMgO NPs // Al. The QLED with 0.05 Cu mole fraction showed higher EL performances, such as maximum luminance of 861 cd/m^2^ and EQE of 3.5%, as compared to the 0.1 Cu mole fraction (269 cd/m^2^, 2.4%). A disparity in EQE between 0.05 versus 0.1 Cu-doped QLEDs was tentatively attributable to a long decaying nature of Cu dopant, by which EL became more sensitive to Cu concentration quenching than PL.
